# Editorial: Radiation-induced and oxidative DNA damages

**DOI:** 10.3389/fchem.2015.00054

**Published:** 2015-08-25

**Authors:** Antonio Monari, Elise Dumont, Chryssostomos Chatgilialoglu

**Affiliations:** ^1^Theory-Simulatio-Modeling Group, Centre National de la Recherche Scientifique, Université de LorraineNancy, France; ^2^École normale supérieure de Lyon and Centre National de la Recherche ScientifiqueLyon, France; ^3^National Center for Scientific Research DemokritosAthens, Greece

**Keywords:** DNA lesions, radiation and UV induced, simulation and modeling, spectroscopies, analytical chemistry

The study of DNA lesions and changes, produced by interaction with UV or ionizing radiation and by the effects of oxidative stress, is a multidisciplinary research field in life sciences that has yelded exciting discoveries in the last few decades. The importance of this study is clearly enhanced by its strong societal impact, because of the connections of DNA lesions with carcinogenetic processes, which lead to the disruption or mismatching of the genetic information. As well, the controlled and selective production of lesions is a promising tool for the development of novel antitumoral strategies and drugs. The multidisciplinary character requires a combined effort from simulation and modeling studies, together with high-level experimental and analytical chemistry, spectroscopy, up to biochemical or cellular biology tools. In this Research Topic, we present a collection of articles focusing on DNA bases photoinduced and radiation damages, on the DNA interactions, on potential drug development and on the effect of radicals on DNA constituents.

The fundamental reactions of DNA constituents are taken into account for instance by the investigation of the potential mutagenic effects of NO_2_ radical to guanine and cytosine base pairs (Cerón-Carrasco et al., 2015) by high level simulation protocols. An ameliorated protocol has also been developed and validated to achieve a better accuracy in the determination of radiation-induced lesions in purine moieties (Terzidis et al., 2015) embedded in double- and single-strand DNA. The improved quantification of purine 5′,8-cyclonucleosides resulting from the cyclization of C5′ radical on DNA has also been tackled (Terzidis and Chatgilialoglu, 2015). The association constant of lipophilic derivatives of the 5′,8-cyclo-2′-deoxyguanosine with 2′-deoxycytidine has been studied by voltammetry NMR techniques (Capobianco et al., 2015).

DNA sensitization by endogenous molecules has also been covered. The oxidative profile of copper phenanthrene chemical nucleases interacting with DNA has been presented (Molphy et al., 2015) together with a variety of biophysical data. Furthermore, Förster Resonant-Energy-Transfer has been used to prove the flexibility and dynamical behavior of short double-strand DNA sequences intercalated by organometallic compounds (Jia et al., 2015). Connected to spectroscopy a very detailed analysis of the excited state potential energy landscape of solvated guanine monophosphate has been reported with high level *ab initio* techniques, together with an analysis of principal photochemical pathways (Altavilla et al., 2015).

Furthermore, two reviews, one on simulation and modeling of DNA lesions (Dumont and Monari, 2015) and the other on stress-induced DNA biomarkers (Nikitaki et al., 2015), help to clearly define the state of the art of this very active scientific field.

This scientific topic is also closely related to the COST in Chemistry Action CM1201 “Biomimetic Radical Chemistry” and in particular to its Working Group 2 devoted to “Models of DNA Damages and Consequences.” The COST Action members have actively participated with most of the contributions derived from the scientific collaborations ongoing in its framework. As such the Research Topic also represents a very nice overview of the effectiveness and added value brought about by the creation of a large multinational scientific network. Furthermore, the financial support of the COST Action CM1201 has allowed the open source publication of exciting scientific contributions from its members.

We believe that the “Radiation Induced and Oxidative DNA Damage” Research Topic nicely illustrates most of the complex and fascinating challenges that scientists working on DNA damages should tackle. At the same time, it highlights the scientific maturity of this field, foreseeing even more exciting perspectives that will undoubtedly blossom in the forthcoming years.

## Conflict of interest statement

The authors declare that the research was conducted in the absence of any commercial or financial relationships that could be construed as a potential conflict of interest.
